# Pathogenic Biofilm Removal Potential of Wild-Type *Lacticaseibacillus rhamnosus* Strains

**DOI:** 10.3390/pathogens12121449

**Published:** 2023-12-14

**Authors:** Gregoria Mitropoulou, Vasiliki Kompoura, Grigorios Nelios, Yiannis Kourkoutas

**Affiliations:** Laboratory of Applied Microbiology and Biotechnology, Department of Molecular Biology and Genetics, Democritus University of Thrace, 68100 Alexandroupolis, Greece; grigoriamitropoulou@gmail.com (G.M.); vickykom20.70@gmail.com (V.K.); gregnelios@hotmail.com (G.N.)

**Keywords:** biofilm removal, *L. rhamnosus*, cell-free supernatant, foodborne pathogen

## Abstract

The emergence of antimicrobial resistance remains one of the greatest public health concerns. Biofilm formation has been postulated as a mechanism of microbial pathogens to resist antimicrobial agents. Lactic Acid Bacteria (LAB) and their metabolites have been proposed to combat bacterial biofilms due to their antimicrobial activity. In this vein, the aim of the present study was to investigate the biofilm removal potential of cell-free supernatants (CFSs) of five wild-type *Lacticaseibacillus rhamnosus* strains, isolated from Greek natural products, in comparison to the commercially available *L. rhamnosus* GG strain, against biofilms formed by common foodborne pathogens (*Salmonella* Enteritidis, *Salmonella* Typhimurium, *Escherichia coli*, *Listeria monocytogenes*, and *Staphylococcus aureus*). The biofilm removal activity of LAB was assessed on a two-day-old mature biofilm using a microtiter plate-based procedure. Both non-neutralized and neutralized CFSs removed biofilms in a concentration-dependent manner. The biofilm removal activity of the non-neutralized CFSs was significantly higher compared to the neutralized CFSs, as expected, with ranges of 60–89% and 30–80%, respectively. The biofilm removal efficiency of *L. rhamnosus* OLXAL-3 was significantly higher among the wild-type *L. rhamnosus* strains tested (20–100% *v*/*v*). In conclusion, our results suggest the great potential of the application of wild-type *L. rhamnosus* strain’ CFSs as effective natural agents against pathogenic bacterial biofilms.

## 1. Introduction

The rising antimicrobial resistance of pathogens poses a worldwide risk to human health, and thus, efficient antimicrobial alternatives are urgently required [1]. Bacterial biofilms, formed by the polymeric metabolites secreted by microbes, are one of the main resistance mechanisms that bacteria utilize to survive against various stresses, including antibiotics, disinfectants, and host defenses. The use of Lactic Acid Bacteria (LAB) to combat bacterial biofilms is a rapidly growing trend. In addition, it has been documented that LAB produce several bioactive molecules, such as organic acids, alcohols, carbon dioxide, diacetyl, hydrogen peroxide, and bacteriocins [2,3], many of which exert powerful antimicrobial activity [2,4]. Generally, most of these compounds are secreted during cultivation in a broth medium following the proliferation of bacterial cells, which is known as a supernatant. Hence, it has been observed that the LAB culture supernatant acts efficiently against bacterial biofilms [5,6,7,8]. Thus, the antimicrobial agents produced by probiotic cell metabolism have recently been proposed as potential candidates for controlling bacterial biofilm formation against foodborne pathogens [9,10,11]. Interestingly, a previous study depicted that probiotics themselves and their dried supernatant could inhibit the formation of dental plaque caused by *Streptococcus mutans* [12].

Bacterial biofilm formation is a consequence of the accumulation and non-reversible attachment of bacterial cells on a biological or non-biological surface, as well as of a body of extracellular polymers (Extracellular Polymeric Substance—ESP), or glycocalyx, that is secreted by the same microorganisms [13,14].

In the food industry, microbial biofilms have been found in dairy, fish, and poultry products, as well as in the production lines, e.g., of manufacturing plants that produce ready-to-eat meals [15]. Members of the species *Salmonella* have been reported to form biofilms on a variety of surfaces and equipment, while *Bacillus cereus*, *Escherichia coli*, *Shigella* spp., and *Staphylococcus aureus* have been isolated during the production process of dairy products [16]. Furthermore, biofilm formation by *E. coli* was observed and isolated from surfaces during the production of cattle-derived meat products [15]. Frequently observed species related to biofilm formation are staphylococci, *Enterobacteriaceae*, and the foodborne pathogen *Listeria monocytogenes*. Therefore, addressing and controlling the formation of microbial biofilms from foodborne pathogens is a challenge for the food industry, where the need to produce safe products is of paramount importance.

Hence, the aim of the present study was to assess the biofilm removal potential of cell-free supernatant (CFS) of five LAB strains belonging to the *Lacticaseibacillus rhamnosus* species, isolated from traditional Greek foods, against biofilms formed by common foodborne pathogens.

## 2. Materials and Methods

### 2.1. Microbial Strains

Five wild-type lactic acid bacteria, the technological and functional properties of which have been described in a previous study [17], isolated from Greek natural products, were used, as shown in Table 1. All strains were grown in de Man, Rogosa, and Sharpe broth (MRS) (10 g/L of Bacteriological peptone, 2 g/L of Dipotassium phosphate, 0.05 g/L of Manganese sulfate, 5 g/L of Sodium acetate, 4 g/L of Yeast extract, 20 g/L of Dextrose, 0.2 g/L of Magnesium sulfate, 8 g/L of Beef extract, 1 g/L of Tween80, 2 g/L of Ammonium citrate) (Condalab, Madrid, Spain) at 37 °C for 24 h. The commercial *Lacticaseibacillus rhamnosus* GG served as the reference strain, since its activity against pathogenic biofilm formation is reported in the literature [18].

*Salmonella enterica* subsp. *enterica* ser. Enteritidis FMCC B56 PT4, *Salmonella enterica* subsp. *enterica* ser. Τyphimurium DSMZ 554, *Escherichia coli* ATCC 25922, *Listeria monocytogenes* NCTC 10527 serotype 4b, and *Staphylococcus aureus* ATCC 25923 were grown in Brain–Heart Infusion (BHI) broth (2 g/L of Glucose, 10 g/L of Enzymatic digest of animal tissues, 5 g/L of Beef heart infusion, 5 g/L of Sodium chloride, 12.5 g/L of Calf brain infusion, 2.5 g/L of Di-sodium hydrogen phosphate) (Condalab, Madrid, Spain) at 37 °C for 24 h.

### 2.2. Preparation of LAB CFS (Cell-Free Supernatants)

An overnight bacterial culture of each isolated wild-type LAB strain (10^9^ cfu/mL) was centrifuged at 8500× *g*, 4 °C for 15 min, and the supernatant (pH 3.8–3.9) was collected. In order to study the neutralized CFSs (pH 7) against bacterial biofilm removal, part of the collected CFSs was neutralized using 5 M of NaOH solution using a pH meter (WTW Ph 330i, WTW, Weilheim, Germany). All CFSs were then sterilized through filtration (0.22 μm, Merck, Darmstadt, Germany) (cell-free supernatant—CFS).

### 2.3. Biofilm Removal Activity

Biofilm removal activity was determined using 24-well polystyrene microtiter plates, according to Koohestani et al. [19], with some modifications. In each well, 1800 μL of Brain–Heart Infusion broth and 200 μL of bacterial suspension were poured after two ten-fold dilutions to obtain a final concentration of 10^6^ logcfu/mL. For the establishment of the bacterial biofilms, the microplates were incubated at 37 °C for 48 h. After incubation, the free (planktonic) cells were discarded and the wells were washed with ¼ Ringer’s (2.25 g/L of NaCl, 0.105 g/L of KCl, 0.12 g/L of CaCl_2_H_12_O_6_, and 0.05 g/L of NaHCO_3_) sterilized solution to remove any weakly attached cells. An optical density (OD) measurement of approximately 1.4 after 48 h suggested a relatively dense biofilm formation, as OD values above 1 generally indicate high cell or biofilm densities (Table S1) [20,21].

In the next step, CFSs (neutralized and non-neutralized) of all LAB strains were gently added to the wells at concentrations of 100%, 80%, 60%, 40%, or 20% and left undisturbed for 1 h at ambient temperature (approximately at 24 °C). Then, the CFSs were vigorously decanted and the wells were washed again with ¼ Ringer’s sterilized solution. The remaining attached bacterial biofilms were stabilized with the addition of 2000 μL of methanol solution and allowed to dry for 5 min. Cell staining was performed using 1% *w*/*v* crystal violet (Sigma-Aldrich, St. Louis, MO, USA) for 30 min, followed by a final wash with distilled H_2_O, in order to remove any excess staining solution. The remaining crystal violet at the bottom of the wells was diluted in ethanol/acetone solution (Chem-Lab NV, Zedelgem, Belgium) at a ratio of 80/20 for 15 min. The solutions were then transferred into clear bottom 96-well microplates to determine the optical absorbance at 540 nm. Wells containing only BHI broth and bacterial suspension without CFSs were used as the negative and positive controls, respectively.

The estimation of the reduction percentage of biofilms exposed to the different CFSs was calculated according to the following equation:

(1) $$\mathrm{Reduction}\;\mathrm{percentage}=\frac{\left(C-B\right)-(T-B)}{\left(C-B\right)}\times 100,$$

where C represents the OD values of positive control wells, B represents the OD values of negative control wells, and T represents the OD values of CFS-treated wells at 540 nm.

### 2.4. Statistical Analysis

All experiments were performed at least in quadruple, and the results were analyzed for statistical significance with analysis of variance (ANOVA). Duncan’s test was used to determine significant differences (coefficients, ANOVA tables, and significance (*p* < 0.05) were computed using Statistica for Windows, v.12.5 (StatSoft, Tulsa, OK, USA)).

## 3. Results and Discussion

This study investigated the biofilm removal potential of cell-free supernatants (CFSs) obtained from five wild-type *Lacticaseibacillus rhamnosus* strains along with the commercially available *L. rhamnosus* GG against biofilms formed by common foodborne pathogens. The efficacies of both non-neutralized and neutralized CFSs were assessed, revealing notable strain-specific activities.

**Figure 1:**
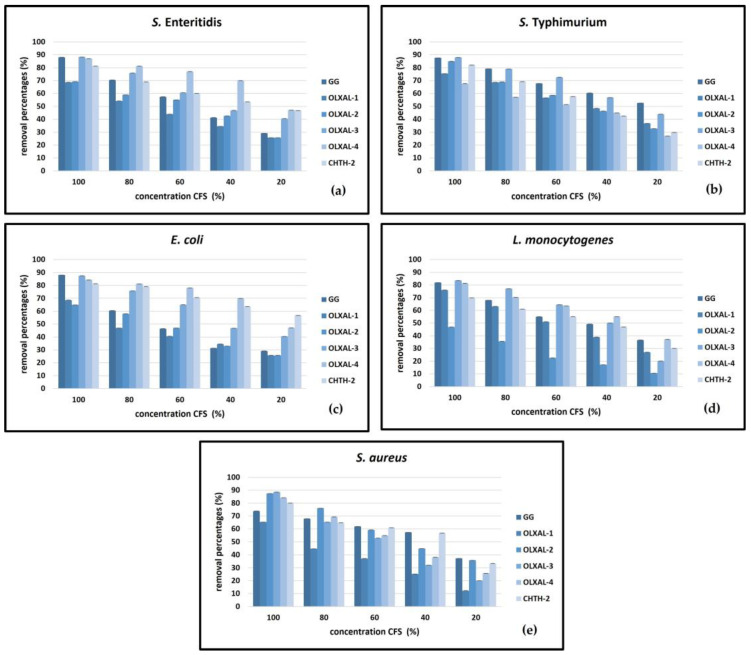
Biofilm removal activity of non-neutralized CFSs of five wild-type *L. rhamnosus* strains in comparison to *L. rhamnosus* GG against (**a**) *S.* Enteritidis, (**b**) *S.* Typhimurium, (**c**) *E. coli*, (**d**) *L. monocytogenes*, and (**e**) *S. aureus*.

**Figure 2:**
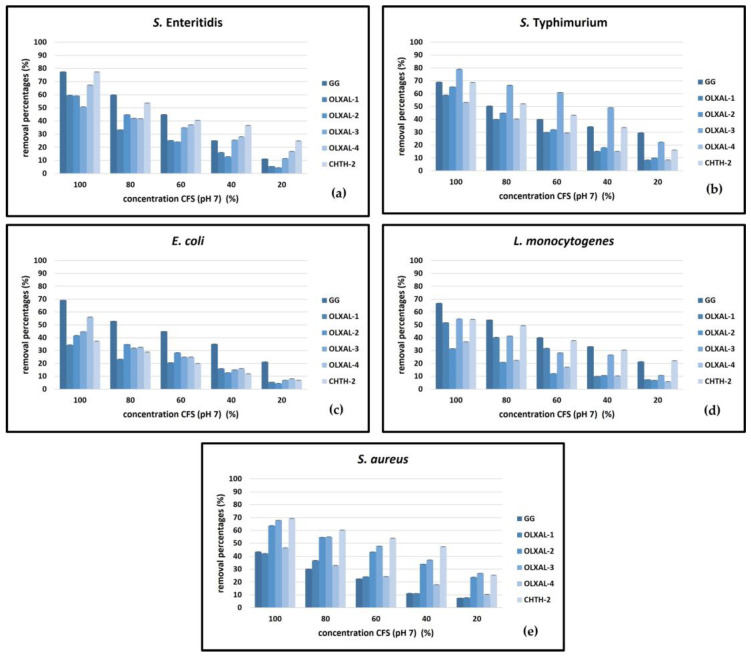
Biofilm removal activity of neutralized (pH, 7) CFSs of five wild-type *L. rhamnosus* strains in comparison to *L. rhamnosus* GG against (**a**) *S.* Enteritidis, (**b**) *S.* Typhimurium, (**c**) *E. coli*, (**d**) *L. monocytogenes* and (**e**) *S. aureus*.

A comparison of CFS effectiveness across pathogens revealed that non-neutralized CFSs exhibited significant biofilm removal activity against *S.* Enteritidis, *S.* Typhimurium, *E. coli*, *L. monocytogenes*, and *S. aureus* (60–89% removal) (Figure 1 and Figure 2). The neutralization of CFSs led to a reduction in efficacy, but still maintained significant removal activity (32–80% removal) compared to the biofilm removal percentages of non-neutralized CFSs (*p* < 0.05).

Among the CFSs of the strains tested, the highest (*p* < 0.05) removal activity was observed with non-neutralized *L. rhamnosus* OLXAL-3 and OLXAL-4 CFSs against *S.* Enteritidis (88–89%), *S. aureus* (84–89%), *E. coli* (84–87%), and *L. monocytogenes* (81–84%). Non-neutralized CFS of OLXAL-3 exhibited strong biofilm removal activity against *S.* Typhimurium (73–88% removal). On the contrary, non-neutralized CFS of OLXAL-4 was not as effective against biofilms of the same pathogen (51–68% removal). Similarly, substantial removal activity of *L. rhamnosus* OLXAL-2 and OLXAL-3 CFSs against *S. aureus* biofilms (87–89% removal) was recorded. Non-neutralized *L. rhamnosus* CHTH-2 CFSs exhibited notable removal efficacy against all pathogens (70–81% removal), but lower (*p* < 0.05) efficacy compared to non-neutralized OLXAL-3 and OLXAL-4 CFSs. However, moderate biofilm removal percentages were observed for non-neutralized *L. rhamnosus* OLXAL-1 CFSs, with ranges of 44–69%, 41–69%, 57–75%, 51–76%, and 37–65% against *S.* Enteritidis, *E. coli*, *S.* Typhimurium, *L. monocytogenes*, and *S. aureus* biofilms, respectively. Effective biofilm removal activity against specific pathogens was observed with non-neutralized *L. rhamnosus* GG CFSs (used as the reference), as removal percentages of 46–88%, 55–82%, 57%–86%, 68–86%, and 62–74% against *E. coli*, *L. monocytogenes*, *S.* Enteritidis, *S.* Typhimurium, and *S. aureus* biofilms, respectively, were recorded. However, these values were significantly lower (*p* < 0.05) than OLXAL-3 non-neutralized CFS activity.

Importantly, noteworthy removal activity against both *S.* Enteritidis and *S.* Typhimurium (35–51% and 37–67%, respectively) with neutralized *L. rhamnosus* OLXAL-3 CFSs was also noted. Similarly, both neutralized *L. rhamnosus* CHTH-2 and OLXAL-2 CFSs demonstrated noteworthy activity against *S. aureus* biofilms (69% and 64% removal, respectively), while neutralized *L. rhamnosus* GG CFSs were effective against *E. coli* and *L. monocytogenes* biofilms (67–69% removal). Of note, after neutralization, the efficacy of OLXAL-4 CFSs on biofilm removal against all pathogens tested was in the range of 37–67%. In contrast, reduced biofilm removal activity against all pathogens tested (34–59% removal) was observed with neutralized *L. rhamnosus* OLXAL-1 CFSs.

Different strains exhibited distinct biofilm removal effectiveness against various pathogens, indicating inherent strain-specific bioactivity variations. CFS neutralization led to reduced activity; yet, certain strains retained notable removal activity, suggesting resilience to pH alterations. Although generally high biofilm removal percentages were recorded with *L. rhamnosus* GG CFSs used as the reference, certain wild-type strains exceeded their efficiency against specific pathogens, suggesting a strain-specific effectiveness, that varied according to the bacterial pathogen.

The above findings are in accordance with previous studies that demonstrated the antimicrobial and biofilm removal activity of LAB CFSs against foodborne pathogens in a concentration-dependent manner [19,22,23]. Notably, it has been recently reported by Nelios et al. [17] that non-neutralized *L. rhamnosus* CFSs exhibited the growth inhibition of foodborne spoilage and pathogenic microorganisms up to 90%, but their activity was significantly reduced after neutralization. Discrepancies in efficacy between the strains might be attributed to variations in metabolite profiles, including the production of organic acids and bacteriocins that might contribute to biofilm removal, suggesting the need for further investigation into the specific mechanisms of action.

The results of the present study are in agreement with previous research by Divyashree et al. [24], in which the effect of non-neutralized CFSs of *Lactobacillus casei* MYSRD 108 and *Lactobacillus plantarum* MYSRD 71 on *Salmonella paratyphi* bacterial biofilms at a 15% *v*/*v* CFS concentration was studied, reporting a removal activity over 75% with the non-neutralized *Lactobacillus casei* MYSRD 108 CFS and 81% with the non-neutralized *Lactobacillus plantarum* MYSRD 71 CFS against *S. paratyphi* biofilms. As expected, neutralized CFSs had significantly lower activity. The greater biofilm removal activity of the non-neutralized CFSs of the two strains was due to the presence of organic acids, according to Divyashree et al. [24]. In another study [23], the non-neutralized CFS of the probiotic strain *Weissella confusa* WM36 resulted in the removal of 95.68% of *Salmonella typhi* biofilm at 20% *v*/*v*, while the non-neutralized CFS of *Weissella viridescens* WM33 removed 66.46% of *Salmonella* Typhimurium biofilms at 15% *v*/*v*. Similarly, Tazehabadi et al. [25] studied the biofilm removal activities of *Bacillus subtilis* KATMIRA 1933 and *Bacillus amyloliquefaciens* B-1895 CFS against *Salmonella enterica* subsp. *enterica* serovar Hadar, *Salmonella enterica* subsp. *enterica* serovar Enteritidis phage type 4, and *Salmonella enterica* subsp. *enterica* serovar Thompson biofilms. The CFS *of Bacillus subtilis* KATMIRA 1933 removed 51.1, 48.3, and 56.9% of the biofilms formed by the *Salmonella* species studied, while the corresponding removal percentages observed with *Bacillus amyloliquefaciens* B-1895 CFS were 30.4, 28.6, and 35.5%, respectively. According to Tazehabadi et al. [24], the activity against biofilms was not associated with low pH and organic acid production, but with the production of subtilisin peptides, as the pH of CFS was approximately 5.85 for both strains.

Abdelhamid et al. [26] studied the activity of non-neutralized CFSs of six probiotic bacteria of the genera *Bifidobacterium* and *Lacticaseibacillus* against biofilms of multi-resistant *E. coli* WW1 and IC2 strains. The CFSs of *B. longum* and *L. plantarum* removed 57.94 and 64.57% of *E. coli* IC2 and *E. coli* WW1 biofilms, respectively. Furthermore, the skim milk CFSs fermented with *L. helveticus* or *L. rhamnosus*, separately, removed 31.52 and 17.68% of *E. coli* IC2 biofilms, respectively, while the corresponding removal percentages recorded with *B. longum* or *L. helveticus* CFS were 70.81 and 69.49%. In a similar study published by Apiwatsiri et al. [27], neutralized and non-neutralized CFSs of *Lactobacillus plantarum* 22F, 25F, and *Pediococcus acidilactici* 72N strains showed significant removal activity against bacterial biofilm strains of *E. coli* resistant to the antibiotic colistin.

According to a study by Shao et al. [28], non-neutralized CFSs of three *Leuconostoc mesenteroides* strains effectively removed *L. monocytogenes* biofilms. *Leuconostoc* species produce bacteriocins, such as mesentericin Y105, which is formed by *Leuconostoc mesenteroides* spp. *mesenteroides*; leucocin A-UAL 187, which is produced by *Leuconostoc gelidum*; carnosin 44A, which is a metabolic product of *Leuconostoc carnosum*; and leuconocin S, which is produced *by Leuconostoc paramesenteroides* [29]. Bacteriocins of *Leuconostoc* species possess significantly high removal activity against *Listeria* biofilms. Moreover, Moradi et al. [30] studied the effect of *L. acidophilus* LA5 and *L. casei* 431 CFS on *L. monocytogenes* biofilms. A greater biofilm removal activity was noted for *L. acidophilus* CFS, and it was found that *L. acidophilus* CFS contains 1.8% lauric acid, which is considered a possible surfactant; yet, the mechanism of action is not fully understood. According to Moradi et al. [30], and in agreement with our results, the action of LAB CFSs in the removal of bacterial biofilms was strain-specific and due to the presence of compounds, such as exopolysaccharides, organic acids, and surfactants.

In a recent study, Koohestani et al. [19] explored the removal activity of *L. acidophilus* LA5 and *L. casei* 431 CFSs against *S. aureus* bacterial biofilms. In accordance with our findings, their results highlighted the significant removal activity of both CFSs tested in a concentration-dependent manner. Of note, the subsequent neutralization of both *Lactobacillus* CFSs resulted in reduced activity, which is in agreement with our results, as well as previous reports [31].

This study demonstrated the significant potential of LAB-derived CFSs in mitigating biofilms formed by various foodborne pathogens. Understanding their mechanisms of action and optimizing their applications could lead to valuable interventions in food safety protocols. Future research could explore additional microbial metabolites and their synergistic effects for enhanced biofilm control strategies.

## 4. Conclusions

A concentration-dependent removal activity (60–89%) of *S.* Enteritidis, *S.* Typhimurium, *E. coli*, *L. monocytogenes*, and *S. aureus* biofilms was observed with the CFSs of the wild-type *L. rhamnosus* strains studied. However, their effectiveness was reduced after neutralization, but still remained significant (32–79% removal activity). Thus, more research is still required to identify the presence of other microbial metabolites, such as bacteriocins, hydrogen peroxide, diacetyl, etc., as well as the expression of specific biofilm genes, and clarify their effect and mechanisms of action in biofilm removal.

Since the food industry is susceptible to the risks associated with biofilm formation and biofilms not only have an impact on food manufacturing operations, but also pose a wide public health risk through the contamination of food products, a better understanding of the mechanisms of their formation in the food chain is essential for developing prevention and control measures.

## Figures and Tables

**Table 1:** Wild-type Lactic Acid Bacteria (LAB) used in the present study.

Isolate Code	Bacterial Species	Source of Isolation
GG (ATCC 53103)	*Lacticaseibacillus rhamnosus*	Human intestines
OLXAL-1	*Lacticaseibacillus rhamnosus*	Olive (fruit)
OLXAL-2	*Lacticaseibacillus rhamnosus*	Olive (fruit)
OLXAL-3	*Lacticaseibacillus rhamnosus*	Olive (fruit)
OLXAL-4	*Lacticaseibacillus rhamnosus*	Olive (fruit)
CHTH-2	*Lacticaseibacillus rhamnosus*	Feta-type cheese

## Data Availability

The data presented in this study are available upon request from the corresponding author. The data are not publicly available due to the restrictions of the funding authorities.

## Supplementary Materials

The following supporting information can be downloaded at: https://www.mdpi.com/article/10.3390/pathogens12121449/s1. Table S1. OD values (540 nm) of positive controls before treatment with *Lacticaseibacillus rhamnosus* strains CFSs.
