# Supplementary material for: Pathogenic Biofilm Removal Potential of Wild-Type Lacticaseibacillus rhamnosus Strains
Source: Pathogens. 2023 Dec 14;12(12):1449. doi: 10.3390/pathogens12121449 (PMC10748307; doi:10.3390/pathogens12121449)
Supplement: Supplementary file 1 [file pathogens-12-01449-s001.zip › pathogens-2719152-SI.pdf]

Supplementary material

Table S1. OD values (540 nm) of positive controls before treatment with *Lacticaseibacillus rhamnosus* strains CFSs.

Microbial species	*L. rhamnosus* strains					
	**GG**	**OLXAL-1**	**OLXAL-2**	**OLXAL-3**	**OLXAL-4**	**CHTH-2**
			**Non-neutralized CFSs**			
***S.* Enteritidis**	1.410±0.003	1.400±0.003	1.401±0.003	1.400±0.003	1.403±0.003	1.411±0.003
***S.* Typhimurium**	1.390±0.003	1.401±0.003	1.411±0.003	1.399±0.003	1.401±0.003	1.400±0.003
E. coli	1.423±0.003	1.422±0.003	1.419±0.003	1.420±0.003	1.421±0.003	1.422±0.003
L. monocytogenes	1.415±0.003	1.416±0.003	1.417±0.003	1.413±0.003	1.418±0.003	1.411±0.003
S. aureus	1.435±0.003	1.432±0.003	1.430±0.003	1.432±0.003	1.433±0.003	1.431±0.003
			**Neutralized CFSs**			
***S.* Enteritidis**	1.403±0.003	1.401±0.003	1.410±0.003	1.402±0.003	1.411±0.003	1.401±0.003
***S.* Typhimurium**	1.399±0.003	1.400±0.003	1.409±0.003	1.403±0.003	1.400±0.003	1.401±0.003
E. coli	1.422±0.003	1.420±0.003	1.421±0.003	1.421±0.003	1.422±0.003	1.423±0.003
L. monocytogenes	1.416±0.003	1.418±0.003	1.416±0.003	1.417±0.003	1.416±0.003	1.417±0.003
S. aureus	1.434±0.003	1.434±0.003	1.432±0.003	1.430±0.003	1.430±0.003	1.432±0.003
